# Case Report: A rare case of concurrence of IgG4-related tubulointerstitial nephritis and renal Amyloid A amyloidosis

**DOI:** 10.3389/fimmu.2025.1672609

**Published:** 2025-11-06

**Authors:** Xiaojuan Zhu, Yueyue Zhu, Wei Wang, Shuang Wang, Jin Xu, Suxia Wang

**Affiliations:** 1 Laboratory of Electron Microscopy, Pathological Center, Peking University First Hospital, Beijing, China; 2 Department of Pathology, Peking University First Hospital, Beijing, China

**Keywords:** IgG4-related disease, IgG4-related lymphadenopathy, IgG4-related tubulointerstitial nephritis, Amyloid A amyloidosis, case report

## Abstract

Immunoglobulin G4-related disease (IgG4-RD) is a systemic immune-mediated fibroinflammatory condition affecting multiple organs. IgG4-related tubulointerstitial nephritis (IgG4-TIN) is the predominant pattern of kidney involvement. Amyloid A (AA) amyloidosis is a systemic amyloidosis that develops secondary to chronic inflammation or infection, most frequently affecting the kidneys. The association between IgG4-RD and AA amyloidosis is rarely reported. Herein, we report a case of a 56-year-old Chinese man presenting with a one-year history of dizziness and fatigue. The clinical evaluation and laboratory findings showed multiple enlarged lymph nodes, elevated serum creatinine, and increased levels of IgG4 and C-reactive protein. A kidney biopsy revealed IgG4-TIN. Furthermore, patchy congophilic amyloid deposits in the interstitium and arteriolar walls were positive for AA protein by immunohistochemical staining. Subsequent cervical lymph node biopsy showed IgG4-related lymphadenopathy. With prednisone and cyclophosphamide treatment, the patient achieved complete remission of renal function and a noticeable decrease in IgG4 and C-reactive protein levels. This is the first reported case to our knowledge of IgG4-related lymphadenopathy, IgG4-TIN, concurrent with renal AA amyloidosis. Clinicians should be aware that AA amyloidosis may occur in patients with IgG4-TIN, warranting further investigation into the underlying mechanisms linking AA amyloidosis to IgG4-RD.

## Introduction

Immunoglobulin G4-related disease (IgG4-RD) is a systemic immune-mediated disease characterized by mass-forming lesions with marked IgG4-positive plasma cells and storiform fibrosis, often with elevated serum IgG4 levels. IgG4-RD can affect multiple organs, including the lymph nodes and kidneys (1). When the kidneys are involved, the predominant manifestation is IgG4-related tubulointerstitial nephritis (IgG4-TIN), which is characterized by the infiltration of abundant IgG4-positive plasma cells, severe tubular atrophy, and storiform fibrosis (2, 3).

Amyloid A (AA) amyloidosis is the extracellular deposition of fibrils of serum amyloid A protein (SAA). It can significantly impair organ function, with the kidneys being the most frequently affected organ. The extent of kidney damage usually defines the prognosis of AA amyloidosis. AA amyloidosis usually occurs in a variety of chronic inflammatory and infectious diseases (4). IgG4-RD, acting as an immune-associated condition, is a rare cause of AA amyloidosis (5, 6), with one report involving the concomitance of ALECT2 amyloidosis and IgG4-RD (7).

We describe a patient who presented with chronic kidney disease and was diagnosed with IgG4-related lymphadenopathy, IgG4-TIN, and concurrent renal AA amyloidosis. To the best of our knowledge, this is the first case report of IgG4-TIN coexisting with renal AA amyloidosis in a patient with IgG4-RD.

## Case presentation

A 56-year-old Chinese man presented with a one-year history of progressive dizziness and fatigue. In October 2023, initial laboratory tests at a local hospital revealed iron deficiency anemia with a hemoglobin (HGB) level of 58 g/L (reference range: 130 – 175 g/L). Besides, the serum creatinine (Scr) level was 127 μmol/L (reference range: 44 – 133 μmol/L), and the albumin level was 21.44 g/L (reference range: 40–55 g/L). The patient was subsequently treated with Ferrous Succinate. However, due to persistent symptoms, a bone marrow biopsy was performed one month later at another hospital. The biopsy demonstrated reactive plasmacytosis, with mature plasma cells accounting for 10.5%. Flow cytometry analysis showed that these plasma cells expressed typical markers including CD38, CD138, and CD19. The cytoplasmic kappa/lambda light chain ratio was 1.67, which falls within the normal range, indicating a polyclonal population without light chain restriction. In addition, the CD20+ B cell population accounted for 0.84%, with a kappa/lambda ratio of 0.89, also within normal limits and suggesting a balanced, polyclonal B cell population. Laboratory results at that time indicated persistent anemia (HGB 60 g/L), elevated Scr (153 μmol/L), and markedly increased IgG levels (57.44 g/L) (reference range: 7.23 – 16.85 g/L). Consequently, the patient received treatment with polysaccharide iron and Roxadustat.

In February 2024, the patient presented to our outpatient clinic with laboratory tests showing HGB 70 g/L, Scr 157.3 μmol/L, IgG 67.9 g/L, IgG4 24.6 g/L (reference range: 0.03 – 2.01g/L), and positive urinary occult blood. Roxadustat treatment was continued. At follow-up in June 2024, the HGB had risen to 80 g/L, the Scr had increased to 273.1 μmol/L, and the 24-h urinary protein was 1.95g (reference range: 0 – 0.15 g/24h).

Following that, the patient was admitted for advanced evaluation. Physical examination revealed a blood pressure of 92/66 mmHg and multiple enlarged lymph nodes (2–3 cm) in the cervical and inguinal areas. There were no proptosis, diplopia, or dry-eye symptoms; the lacrimal glands were non-palpable and non-tender. High-resolution neck ultrasonography showed normal echotexture and size of both parotid and submandibular glands, without hypoechoic nodules or hyper-vascularity. Laboratory tests showed worsening renal function (Scr 243.4 μmol/L), elevated Urea (18.12 mmol/L) (reference range: 1.8 – 7.1 mmol/L), hypoalbuminemia (24.8 g/L), and elevated total protein (113.9 g/L) (reference range: 65–85 g/L). The systemic inflammatory response was mild, with a C-reactive protein (CRP) level of 72.03 mg/L (reference range: 0–8 mg/L). HGB was 69 g/L, while white blood cell and platelet levels remained within normal ranges. Indirect immunofluorescence revealed high-titer ANA (1:10,000) (reference range < 1: 100). The patient’s anti-SSA antibody level was 21, which is above the reference range of <20. Serum antineutrophilic cytoplasmic antibodies (ANCA) testing was negative by both indirect immunofluorescence and ELISA. Besides, anti-GBM antibodies, anti-cyclic citrulline polypeptide antibody, antistreptolysin O, and cryoglobulin were all negative. Viral serologies for HBV, HCV, and HIV were also negative. Additionally, immunofixation electrophoresis of serum and urine was negative for monoclonal immunoglobulin. Serum complement C3 and C4 levels were not obtained at the time of presentation.

**Figure 1:**
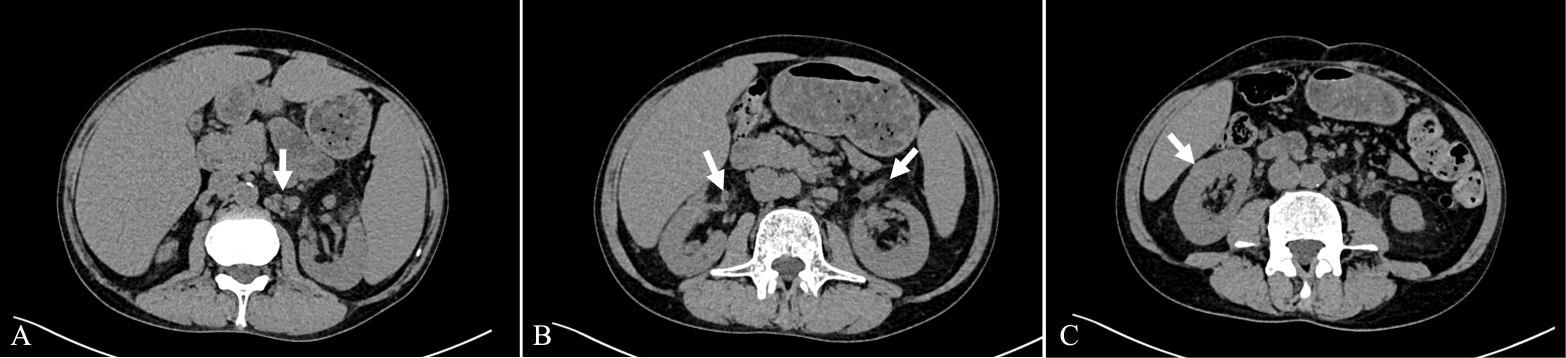
Axial view of the CT scan. **(A)** Enlarged lymph nodes in the retroperitoneal region. **(B)** Exudation around the renal pelvis. **(C)** Isodense nodular lesion under the renal capsule.

Computed Tomography (CT) scans revealed splenomegaly and multiple enlarged lymph nodes in the mediastinum, bilateral axillae, and the retroperitoneal region (**Figure 1A**). Both kidneys appeared normal in size and shape; however, exudation was observed around the renal pelvis (**Figure 1B**). Additionally, an isodense nodular lesion measuring approximately 0.5cm in diameter was observed under the renal capsule in the mid-portion of the right kidney (**Figure 1C**).

**Figure 2:**
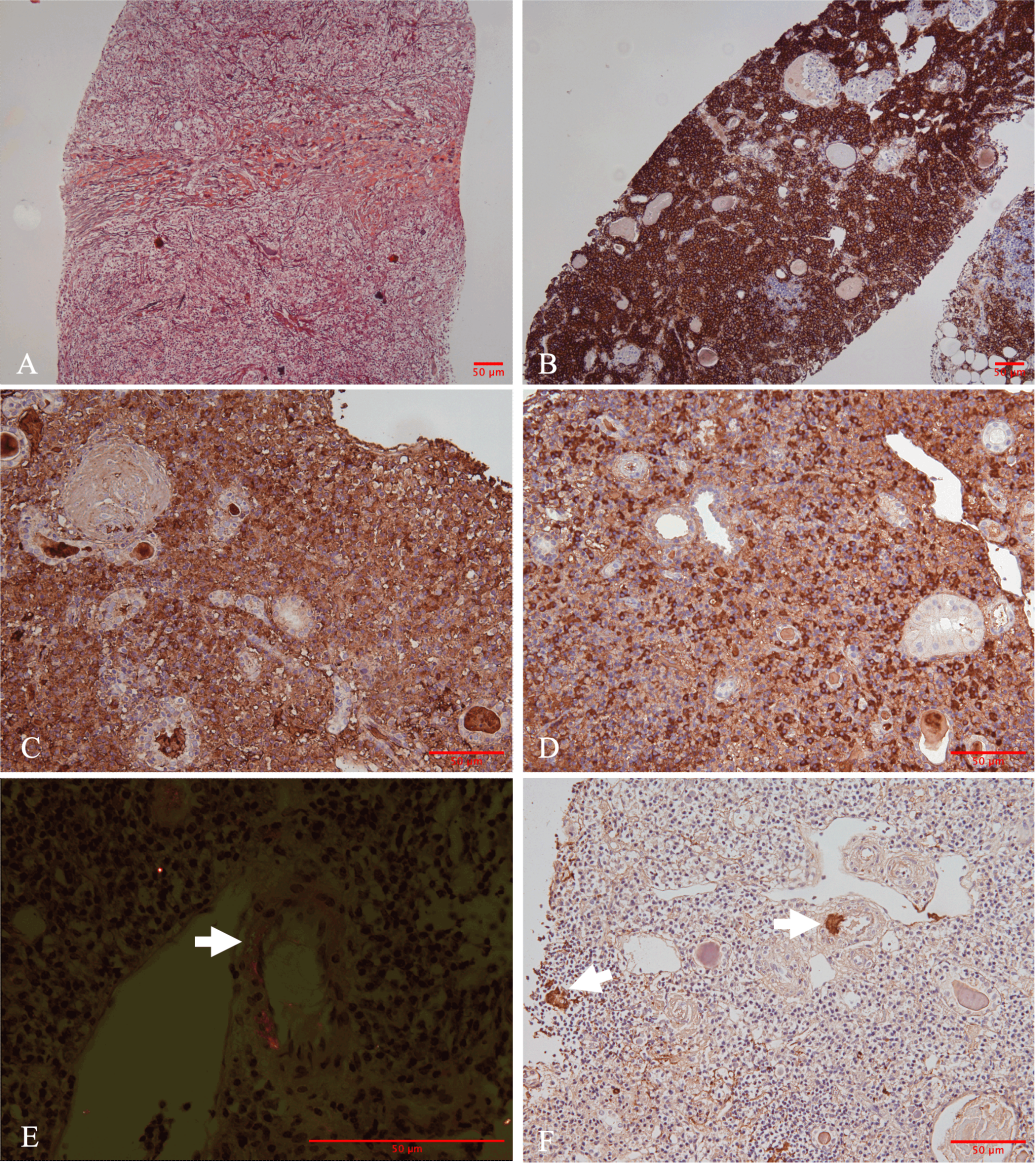
Morphologic and immunohistochemical findings in the renal biopsy. **(A)** Marked storiform pattern of interstitial fibrosis (Jones methenamine silver stain, ×100). **(B)** Immunohistochemically, plasma cells were positive for CD138. **(C)** Immunoglobulin G (IgG) and **(D)** IgG4. The hotspots revealed an IgG4/IgG ratio in 60%. **(E)** Positive Congo red-stained material with apple-green birefringence under Congo red stain under polarized light was identified in the interstitium and arteriolar walls (straight arrows). **(F)** Amyloid deposits were positive for Amyloid A (straight arrows) [**(B)** immunohistochemistry, ×100; **(C-D, F)** immunohistochemistry, ×200; **(E)** polarized light staining, ×400].

A percutaneous biopsy of the right kidney was performed. Light microscopy showed nine glomeruli, including eight globally sclerosed and one fibrotic crescent. The single fibrotic crescent involved <5% of all glomeruli; no fibrinoid necrosis, basement-membrane rupture, or tuft collapse was present. The renal interstitium demonstrated dense interstitial infiltration composed of lymphocytes, eosinophils, and plasma cells, accompanied by severe tubular atrophy and storiform fibrosis (**Figure 2A**). No obliterative phlebitis was identified in any of the sections examined. Immunohistochemical staining demonstrated that CD138-positive plasma cells predominantly infiltrated the interstitium (**Figure 2B**). IgG4-positive plasma cells were approximately 100 cells per HPF with an IgG4/IgG ratio >60% (**Figures 2C, D**). Additionally, patchy congophilic deposits were found in the interstitium and arteriolar walls, exhibiting apple-green birefringence under polarized light (**Figure 2E**). The amyloid deposits were positive for AA protein by immunohistochemical staining (**Figure 2F**). Immunofluorescence study showed that there are no IgG, IgA, IgM, and kappa and lambda light chains in either glomeruli or tubulointerstitium.

**Figure 3:**
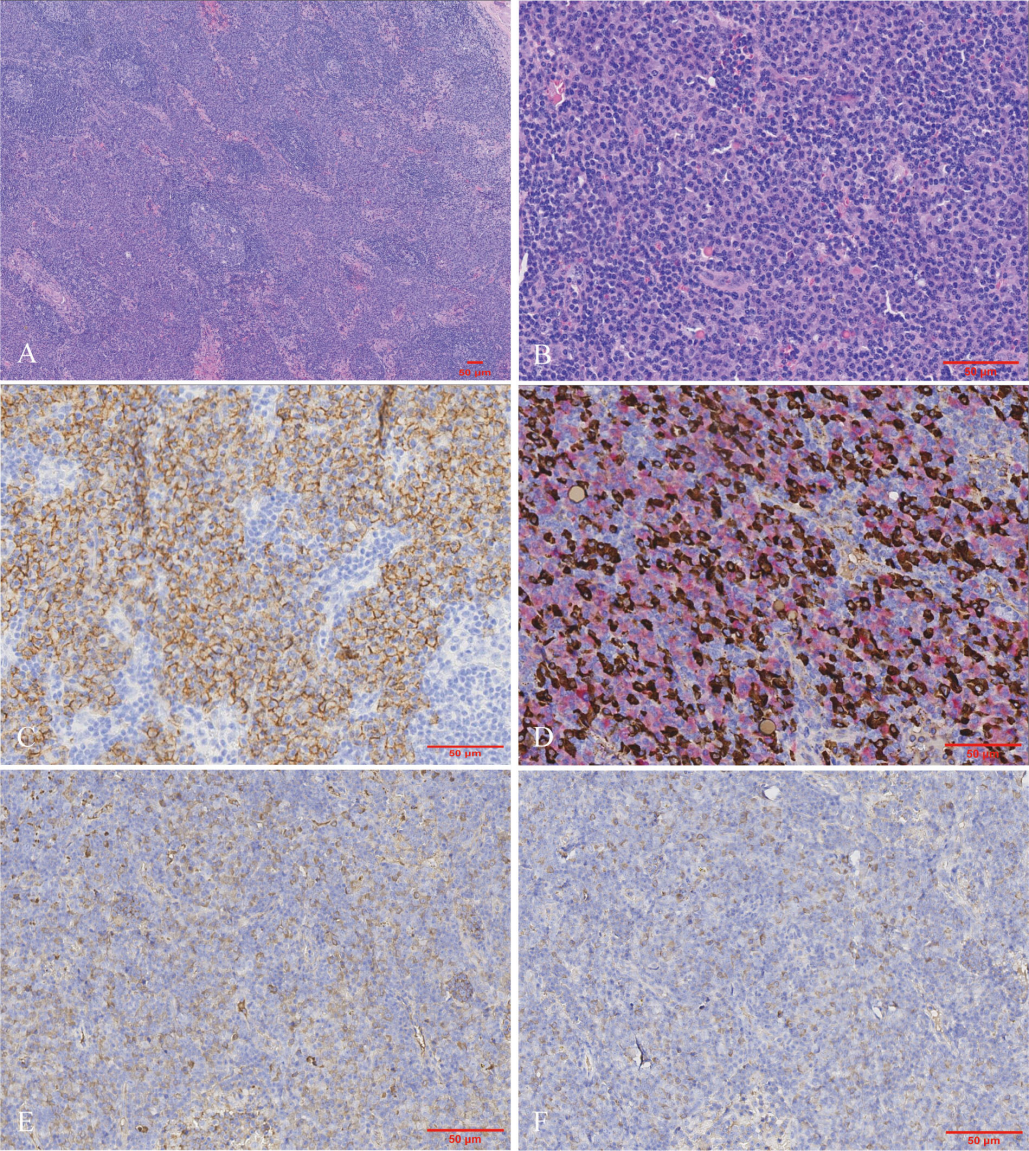
Morphologic and immunohistochemical findings in the lymph node biopsy. **(A)** The nodal architecture was intact, showing hyperplastic and regressed follicles. The interfollicular zone appeared amphophilic staining (Hematoxylin and eosin, ×40). **(B)** The interfollicular expansion pattern was characterized by the proliferation of plasma cells and sparsely distributed eosinophils (Hematoxylin and eosin, ×200). Immunohistochemically, the plasma cells were positive for **(C)** CD138 as well as **(D)** kappa (highlighted in red) and lambda (highlighted in brown) light chains via double-staining techniques. Numerous plasma cells show **(E)** IgG and **(F)** IgG4 expression. Hotspots revealed the IgG4/IgG ratio was up to 70% [**(C–F)** immunohistochemistry, ×200].

Subsequently, a cervical lymph node biopsy was performed. The nodal architecture was preserved, with lymphoid follicles distributed sparsely. Both hyperplastic follicles and follicles exhibiting varying degrees of regressive changes were observed (**Figure 3A**). The interfollicular region exhibited an abundance of plasma cell infiltration, increased high endothelial venules, and scattered eosinophilic infiltrates (**Figure 3B**). Immunohistochemical staining demonstrated that the CD138-positive plasma cells prominently infiltrated the interfollicular spaces (**Figure 3C**). No restriction of light chain expression was detected in the infiltrating plasma cells (**Figure 3D**). IgG4-positive plasma cells were approximately 200 cells per HPF with an IgG4/IgG ratio >70% (**Figures 3E, F**).

**Figure 4:**
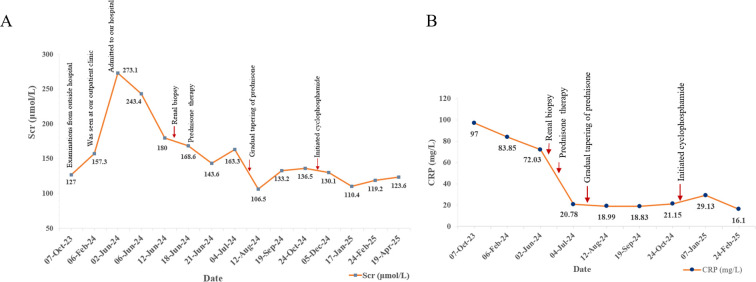
Changes of Scr **(A)** and C-reactive protein **(B)** levels during follow-up.

Following the comprehensive evaluation, a diagnosis of IgG4-RD involving the kidneys and lymph nodes, concurrent with renal AA amyloidosis, was established. The patient commenced treatment with oral prednisone at an initial dose of 40 mg daily to induce remission and manage systemic inflammation. The prednisone dose was gradually tapered over the subsequent months. In October 2024, daily cyclophosphamide (50 mg) was introduced to further suppress disease activity. As of the latest follow-up in April 2025, the patient remained on a low-dose maintenance regimen of prednisone (10 mg and 5 mg on alternating days) and has completed 6 months of cyclophosphamide treatment with stable renal function. After two weeks of prednisone therapy in July 2024, the 24-h urinary protein decreased to 1.16g, reflecting a partial improvement in renal involvement. Scr reduced from a peak of 273.1 μmol/L in June 2024 to approximately 120 μmol/L by April 2025. Furthermore, CRP levels declined from 97 mg/L to 16.1 mg/L, indicating effective management of systemic inflammation. Indicators of renal function (Scr) and systemic inflammation (CRP) are depicted (**Figure 4**). Although serological and renal function improvements were documented, no follow-up CT was obtained in view of adequate clinical response and concern for additional radiation exposure. The patient continues to be monitored and maintains stable renal function under follow-up care.

## Discussion

IgG4-RD is a systemic immune-mediated disease characterized by mass-forming lesions with marked IgG4-positive plasma cells and storiform fibrosis, often with elevated serum IgG4 levels (1). Patients with different organ involvement exhibit heterogeneous and nonspecific symptoms, which can pose diagnostic challenges. In our case, the patient presented with dizziness and fatigue for more than one year from the onset of clinical symptoms to diagnosis. The diagnosis was not made until persistently elevated serum creatinine levels and enlarged lymph nodes were observed. After a comprehensive series of examinations, including imaging and pathological assessments of kidney and lymph node biopsies, the patient was ultimately diagnosed with IgG4-related lymphadenopathy and IgG4-related tubulointerstitial nephritis (IgG4-TIN) according to the 2020 IgG4-RD diagnostic guidelines (8). He did not meet the 2019 ACR/EULAR classification criteria due to the presence of exclusionary features such as positive anti-SSA antibodies and splenomegaly. However, in this case, the constellation of clinical presentation, elevated serum IgG4, characteristic histopathological findings, and favorable response to glucocorticoid therapy strongly supported the diagnosis of IgG4-RD. This underscores the importance of integrating clinical judgment, serological findings, and histopathological evidence – especially in cases where atypical features may coexist – rather than relying solely on classification criteria designed primarily for research and trial enrollment (9).

IgG4-RD can affect nearly any organ and often presents with multiorgan involvement, including the kidney and lymph nodes (10). Kidney involvement is referred to as IgG4-related kidney disease (IgG4-RKD), presenting as IgG4-TIN, IgG4-related glomerular lesions (particularly membranous nephropathy), and obstructive uropathy, mainly secondary to retroperitoneal fibrosis. IgG4-TIN is the most prevalent form of kidney involvement in IgG4-RKD (3). *Buglioni* et al. (11) reported that extrarenal involvement was present in 79% of the IgG4-RKD cases. Lymphadenopathy occurs in approximately 30–60% of patients with IgG4-RD. Lymphadenopathy attributable to IgG4-RD has been termed IgG4-related lymphadenopathy (12, 13). In our patient, he was also diagnosed with coexisting IgG4-related lymphadenopathy. Notably, in cases with lymph node involvement, the hallmark histopathological feature, such as storiform fibrosis, is frequently absent. Alternatively, the predominant features are an increased presence of polytypic IgG4-positive plasma cells and a variety of reactive lymphadenopathies (14). The same pathological changes were observed in our patient, characterized by massive plasma cell infiltration and absence of storiform fibrosis.

Furthermore, the kidney biopsy also revealed the presence of AA amyloidosis in our patient. AA amyloidosis is a rare systemic complication that often results from chronic inflammatory and infectious disorders, and is characterized by the extracellular deposition of fibrils derived from the SAA protein. The kidneys are the major affected organ, and renal damage usually dictates the prognosis of AA amyloidosis (4). To date, only two cases presented with the concomitance of AA amyloidosis and IgG4-RD, in which IgG4-RD mainly affected lymph nodes, and AA amyloidosis involved the liver and glomeruli of the kidney, respectively (5, 6). There has also been a case report in the literature of IgG4-RD coexisting with ALECT2 amyloidosis (7). The current case exhibited a different pattern from the reported ones, which presented with the concurrence of IgG4-TIN and AA amyloidosis involving renal arterioles and tubulointerstitium. Hence, our case expands the disease spectrum of IgG4-RKD. When diagnosing IgG4-RD, clinicians should pay attention to the possibility of concurrent renal amyloidosis, including both AA amyloidosis and ALECT2 amyloidosis.

IgG4-RD is considered one of the causes of AA amyloidosis (4). However, the underlying pathophysiologic mechanism remains poorly defined. One-third of patients in a study of 28 patients with IgG4-RD had high serum AA levels (15). Both CRP and SAA act as acute-phase proteins, with their blood levels increasing in response to inflammation. Multiple studies have indicated a high correlation between CRP and SAA levels (15, 16). Notably, CRP levels are significantly elevated in patients with IgG4-RD (17). In the two previously reported cases of IgG4-RD associated with secondary AA amyloidosis, elevated levels of CRP were also observed (5, 6). Therefore, we speculated that in our case, the elevated CRP level not only indicated the severity of the inflammatory response in these patients but also suggested the presence of high levels of SAA in blood, which finally led to the occurrence of AA amyloidosis. In this case, the patient exhibited both IgG4-TIN and AA renal amyloidosis, which may suggest a potential pathophysiological link between them. Sustained stimulation of the chronic inflammatory state of IgG4-RD leads to the synthesis and deposition of amyloid precursor proteins. Then, the deposition of AA amyloid material may further exacerbate injury to the renal interstitium, creating a vicious cycle.

A prompt response following glucocorticoid therapy is a characteristic feature of IgG4-RD patients who have abundant IgG4-positive plasma cell infiltration (18). In our patient, treatment with prednisone and cyclophosphamide significantly improved renal function and inflammation status. Previous evidence indicates that the combination of glucocorticoids and cyclophosphamide reduces the risk of relapse in IgG4-RD patients with internal organ involvement. This provides a rationale for the therapeutic strategy adopted in our case (19). Glucocorticoids and immunosuppressants exert their immunosuppressive effects and can effectively control the inflammatory response associated with IgG4-RD, thereby reducing tubular injury. Additionally, these therapies may also have a beneficial effect on AA amyloidosis by reducing the underlying chronic inflammation, which could potentially slow the deposition of amyloid proteins.

Although the treatment outcome in this case was favorable, the prognosis for patients with IgG4-TIN combined with AA amyloidosis remains unclear, and long-term monitoring and management may be necessary. Future studies should focus on the pathogenesis of this disease combination, explore the specific role of the inflammatory response within this, identify optimal treatment strategies, and investigate factors influencing the long-term prognosis to provide further guidance for clinical practice.

This case study has some limitations. The limited quantity of available tissue specimens constrained the feasibility of comprehensive mass spectrometry and electron microscopy analyses. Although hypocomplementemia is common in IgG4-related tubulointerstitial nephritis, C3 and C4 values were unavailable in our patient.

This case enhances our understanding of the clinical spectrum of IgG4-RD, providing a new perspective and evidence for investigating the potential association between IgG4-RD and AA amyloidosis. Furthermore, our case is expected to offer important reference for optimizing clinical diagnostic and treatment strategies, as well as for elucidating the underlying pathogenesis of this rare related condition.

## Data Availability

The raw data supporting the conclusions of this article will be made available by the authors, without undue reservation.
